# Eye contact boosts the reflexive component of overt gaze following

**DOI:** 10.1038/s41598-020-61619-6

**Published:** 2020-03-16

**Authors:** Mario Dalmaso, Giada Alessi, Luigi Castelli, Giovanni Galfano

**Affiliations:** Department of Developmental and Social Psychology University of Padova, Padova, Italy

**Keywords:** Saccades, Social neuroscience, Human behaviour

## Abstract

Establishing eye contact with an individual can subsequently lead to a stronger gaze-mediated orienting effect. However, studies exploring this phenomenon have, so far, only assessed manual responses and focused on covert attention – namely, without eye movements. Here, in two experiments, we explored for the first time whether eye contact can also impact on overt attention in an oculomotor task. This approach has two main advantages, in that it relies on more sensitive, online measures of attention allocation and it better mimics real life settings. Participants performed leftwards and rightwards eye movements in response to a central cue. Furthermore, a task-irrelevant central face established – or not – eye contact with the participant, and then averted its gaze either leftwards or rightwards. Hence, eye movement direction was either congruent or incongruent with that of the gaze stimulus. In both experiments, a gaze following behaviour emerged – specifically, smaller saccadic latencies and a greater accuracy emerged on congruent than on incongruent trials – but its magnitude was not modulated by eye contact. However, in Experiment 2 – in which the different eye contact conditions were presented intermixed rather than blocked, thus making eye contact contextually salient – eye contact led to an overall decrement of saccadic latencies and enhanced the reflexive component of gaze following. Taken together, these results provide novel evidence indicating that eye contact can impact on both eye movements programming and overt gaze following mechanisms, at least when eye contact is made contextually salient.

## Introduction

In everyday social interactions, meeting the eyes of others can inform us regarding several social dimensions of our conspecifics, such as their beliefs, intentions or emotions^1,2^. The relevance of eye stimuli for human beings finds also support under an anatomical perspective. Indeed, we are the only primate species with a white sclera^3,4^, which would allow for a rapid detection of others’ eye-gaze direction^5^. Importantly, understanding whether another individual is looking at us is an essential ability to successfully navigate within social environments, since it may activate the execution of adequate approach-avoidance behavioural responses. Moreover, a bulk of studies has reported that establishing eye contact with others can also shape our physiological and cognitive responses profoundly, and different theoretical frameworks and models have been pushed forward to explain the impact of eye contact on human behaviour^6–10^. The overall picture emerging from this literature suggests that eye contact episodes are typically associated with incremental effects. For instance, faces establishing eye contact with an observer are associated with enhanced arousal levels, are better memorised and improve social categorisation as compared to averted-gaze faces^11–13^. More related to the present work, eye contact can also impact on different mechanisms underlying attention allocation over space. In this regard, it has been observed that direct-gaze faces presented in peripheral spatial locations can more strongly capture attention as compared to both averted-gaze and closed-eye faces, a result reported with both manual^14,15^ and oculomotor measures^16–18^. In a similar vein, when presented at fixation, direct-gaze faces can also hold attention more effectively as compared to other eye-gaze conditions. Also in this case, studies using both manual^19,20^ and oculomotor measures^21,22^ supported this notion.

Other studies have reported that eye contact episodes can also shape the so-called gaze cueing effect. In the typical paradigm for assessing this effect, participants are asked to manually respond to a peripheral target preceded by an uninformative averted-gaze face presented at fixation. Usually, reaction times are faster for targets presented at gazed-at locations than at nongazed-at locations, thus suggesting an influence on covert attention processes^23–26^. Intriguingly, the magnitude of this gaze cuing effect appears to be magnified if the face providing the gaze cue has previously established eye contact with the observer. An initial hint about this phenomenon can be found in a study employing two real faces simultaneously^27^. More precisely, at the beginning of each trial, one face gazed at the participant while the other one looked elsewhere. Afterwards, one of the two faces averted its gaze either towards a target stimulus or toward an empty location and participants provided a manual response to decide whether the averted-gaze face was looking at the target or not. The authors reported a trend suggesting smaller reaction times for faces who made eye contact with the participant and then looked at the target, as compared to the other conditions. A more recent study has reached a similar conclusion by employing a supraliminal avatar face^28^ (Experiment 1). In this case, the face either established eye contact with the participant or not, and then looked either leftwards or rightwards. Subsequently, a peripheral target requiring a manual response appeared either at the gazed-at location or the nongazed-at location. A greater gaze cueing effect emerged when the face had initially displayed eye contact. An analogous result emerged even with a humanoid robot providing non-predictive gaze cues^29^ (Experiment 1). More precisely, participants sat in front of a robot that could either establish eye contact with them or not. Then, the robot turned its head either leftwards or rightwards and a target randomly appeared on one of two peripheral screens. In line with the previous works, manual response times showed a reliable gaze cuing response only after eye contact episodes. The rationale behind the studies discussed above is that the enhanced gaze cueing effect after eye contact may have evolved to facilitate interpersonal communication and social coordination (see also^6,30^). Indeed, receiving eye contact from other individuals could indicate their intention to interact with us. Thus, in turn, it might be advantageous to monitor the signals coming from these individuals – including their eye gaze direction – more properly, in order to eventually provide an appropriate response behaviour. Notably, a common feature of the three aforementioned studies on eye contact and gaze cueing^27–29^ is that they explored the impact of eye contact on gaze cueing only by looking at *covert* attention mechanisms, since participants were asked to avoid eye movements during the task, and to provide a manual response to the target. However, our everyday social interactions are characterised by an intensive use of eye movements that we typically perform to keep track of the multiple stimuli provided by our conspecifics^31^. Hence, an experimental approach based on *overt* oculomotor tasks has an intrinsic, higher ecological validity with respect to classic manual response tasks. Moreover, oculomotor measures are a more direct index of attentional allocation over space as compared to manual reaction times used in covert attention studies, which represent the final product of several processing stages, and they can also provide richer data sets including both temporal and spatial information. In sum, the study of eye movement behaviour in response to social stimuli is of great interest since it might reveal a more fine-grained and ecologically-valid picture about the impact of eye contact on social attention mechanisms with respect to covert attention studies.

The present study provides the first attempt to investigate whether the enhanced covert social orienting observed after eye contact is also reflected in *overt* attentional mechanisms revealed through oculomotor parameters. If confirmed, this would suggest that the eye contact effects on social orienting extend beyond those reported through the gaze-cueing task^27–29^. For this reason, here we employed a well-known paradigm specifically developed to investigate the impact of eye-gaze stimuli on overt orienting^32^. This paradigm consists in asking participants to perform either a leftwards or a rightwards eye movement (i.e., a saccade) depending on the value (e.g., the specific colour) assumed by a central instruction cue. In addition, a task-irrelevant central face can look either leftwards or rightwards. In so doing, the direction of the instructed saccade can be either congruent or incongruent with that of the gaze stimulus. Typically, smaller saccadic latencies and a greater accuracy emerge on congruent than on incongruent trials, indicating the presence of a gaze following behaviour^33–38^. Here, in two experiments we employed a modified version of this paradigm in which the task-irrelevant central face could either establish eye contact with participants or not before they were prompted to execute a saccadic eye movement. In Experiment 1, eye contact conditions (i.e., eye contact vs. no eye contact) were presented in two distinct blocks, in line with previous works^29,39,40^, which has the main advantage to collect a response that is not eventually affected by the presence of other types of stimuli. Based on the previous studies on eye contact and covert attention, we expected a magnified gaze following behaviour when the central face had previously established eye contact with the participant as compared to the condition in which no eye contact had been established. The adoption of an oculomotor paradigm enabled us to conduct more thorough analyses on several possible markers of the investigated phenomenon.

## Experiment 1: Blocked presentation of eye contact

### Participants

Based on the three previous studies that explored eye contact effects on covert gaze-mediated orienting (N = 10 in^27^; N = 22 in^28^; N = 34 in^29^), we aimed to test around 25 participants. We stopped at N = 29 (*Mean age* = 23 years, *SD* = 2.38, 6 males) at the end of a block of testing session. All participants had normal or correct-to-normal vision, were naïve to the purpose of the experiment and provided a written informed consent. All the methods used in all experiments performed during the present study were performed in accordance with the relevant guidelines and regulations of the local research ethics committee. The study was approved by the Ethics Committee for Psychological Research at the University of Padova and conducted in accordance with the Declaration of Helsinki.

### Apparatus

Eye movements were recorded monocularly through an EyeLink 1000-Plus (SR Research) at 1000 Hz. A chinrest was placed 70 cm away from a 24-inch monitor (1280 ×1024 pixels, 120 Hz). Stimuli presentation was controlled through Experiment Builder (SR Research).

### Stimuli and procedure

One male and one female avatar face were created through DAZ 3D software (https://www.daz3d.com/). For each face we had different versions displaying closed eyes, direct gaze, and averted gaze (upwards, leftwards, and rightwards). Avatar faces were employed in order to present participants with well-controlled stimuli with adequate ecological validity.

**Figure 1:**
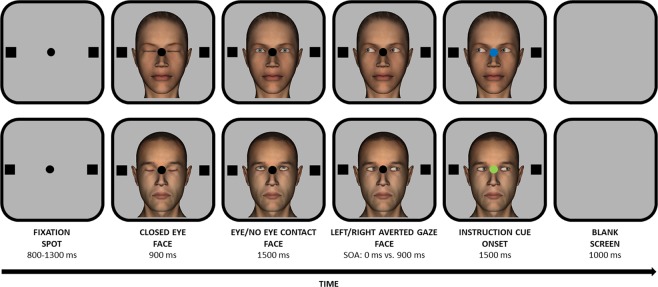
Examples of trials employed in both experiments. The upper panel depicts the female avatar face making eye contact with the observer. The lower panel depicts the male avatar face making no eye contact with the observer. Please note that stimuli are not drawn to scale.

Colour background was set to grey (R = 180, G = 180, B = 180). For each participant, a calibration/validation procedure was performed and then the experiment started. Examples of trials are depicted in Fig. 1. Each trial started with a central black fixation spot (diameter: 0.5°) flanked by two black placeholders (side: 0.9°) placed 9.7° leftwards and rightwards with respect to the central spot. A 500-Hz tone was also played to inform participants of trial initiation. Participants looked at the central spot and the trial continued only if they maintained their eyes on this spot for a variable time period (range: 800–1300 ms; steps: 100 ms), assessed through a gaze-contingent trigger (diameter of the invisible boundary: 4°). If they failed, after 10 s, a visual feedback appeared for 2000 ms, the trial was aborted and recycled at the end of the block, and a new calibration/validation procedure was performed by the experimenter. Otherwise, in case of successful fixation, a central face (10° width × 13° height) with closed eyes appeared for 900 ms. Then, depending on block condition, the same face was presented for 1500 ms with either direct gaze (i.e., eye contact condition) or gaze averted upwards (i.e., no eye contact condition). Then, the face looked either leftwards or rightwards. After a stimulus onset asynchrony (i.e., SOA) of either 0 ms (i.e., simultaneously) or 900 ms, the central spot turned either blue or green (i.e., cue onset) for 1000 ms. If the instruction cue was blue or green, participants were instructed to make a leftwards or a rightwards saccade, respectively. The association between colour and saccade direction was counterbalanced across participants. Finally, a blank screen appeared for 1000 ms. On half of the trials, the direction of the instructed saccade matched the direction indicated by the gaze of the face stimulus (i.e., congruent trials), on the other half there was no matching (i.e., incongruent trials). For each eye-contact condition (i.e., eye contact vs. no eye contact) there was a practice block composed of 5 trials followed by an experimental block composed of 160 trials (i.e., 320 experimental trials in total). A break was allowed every 80 trials. Before each block and after each break, the experimenter performed a drift checking procedure. Block order was counterbalanced across participants.

At the end of the oculomotor task, the participants were administered the Autism-spectrum Quotient (AQ^41^). Indeed, since there is a documented link between eye gaze processing and autistic-like traits^42–45^, we explored whether this link also embraced overt gaze following behaviour.

The whole experimental procedure (oculomotor task and AQ) lasted about 60 minutes.

### Results

#### Data handling

Eye movements with a velocity and acceleration exceeding 30°/s and 8000°/s^2^, respectively, and with a minimum amplitude of 2°, were defined as saccades. On each trial, we extracted the first saccade performed after instruction cue onset. Saccades with blinks were discarded and not further analysed (2.23% of trials). Saccadic directional errors, namely saccades not performed towards the location indicated by the instruction cue, were analysed separately (5.98% of trials). Correct saccades with a latency smaller than 80 ms or greater than 800 ms were considered outliers and discarded from the analyses (0.6% of trials). Inspired by previous studies^33,46,47^, a host of different analyses were carried out in order to explore in detail the potential impact of eye contact on gaze following behaviour.

#### Saccadic latencies

If eye contact increases overt gaze following, then we should expect a greater difference between congruent and incongruent trials after eye contact episodes. Moreover, this difference was expected to be greater at the 0-ms SOA than at the 900-ms SOA. Indeed, previous studies indicated that the influence of social variables on social orienting may decay with time^21,38,48,49^.

**Figure 2:**
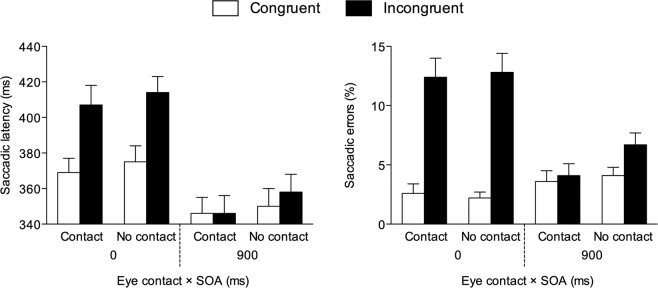
Experiment 1: Saccadic latencies and errors observed on congruent and incongruent trials as a function of SOA and eye contact. Bars are SEM.

Mean saccadic latencies were analysed through a repeated-measures ANOVA with congruency (2: congruent vs. incongruent), SOA (2: 0 vs. 900 ms) and eye contact (2: eye contact vs. no eye contact condition) as within-participant factors. The main effect of congruency was significant, *F*(1, 28) = 59.797, *p* < 0.001, *η*^2^_*p*_ = 0.681, due to shorter latencies on congruent (*M* = 360 ms, *SE* = 8.55) than on incongruent (*M* = 381 ms, *SE* = 9.29) trials, as well as the main effect of SOA, *F*(1, 28) = 229.799, *p* < 0.001, *η*^2^_*p*_ = 0.891, due to shorter latencies at the 900-ms SOA (*M* = 350 ms, *SE* = 9.07) than at the 0-ms SOA (*M* = 391 ms, *SE* = 8.79). The main effect of eye contact was not significant, *F*(1, 28) = 2.370, *p* = 0.135, *η*^2^_*p*_ = 0.078. The congruency × SOA interaction was significant, *F*(1, 28) = 46.782, *p* < 0.001, *η*^2^_*p*_ = 0.626. Paired t-tests indicated that latencies were shorter on congruent than on incongruent trials at the 0-ms SOA, *t*(28) = 8.705, *p* < 0.001, *d* = 1.617, but not at the 900-ms SOA, *t*(28) = 1.405, *p* = 0.171, *d* = 0.261. All the other results, including the theoretically-relevant congruency × SOA × eye contact interaction, were non-significant (*F*s < 1, *p*s > 0.348; see Fig. 2).

#### Saccadic errors

In line with latency analyses, a greater difference between congruent and incongruent trials was expected after eye contact episodes, and in particular at the 0-ms SOA. The percentage of saccadic directional errors was therefore analysed in the same manner as latencies.

The main effect of congruency was significant, *F*(1, 28) = 30.859, *p* < 0.001, *η*^2^_*p*_ = 0.524, due to fewer errors on congruent (*M* = 3.14%, *SE* = 0.49) than on incongruent (*M* = 8.99%, *SE* = 1.13) trials, as well as the main effect of SOA, *F*(1, 28) = 28.153, *p* < 0.001, *η*^2^_*p*_ = 0.501, due to fewer errors at the 900-ms SOA (*M* = 4.63%, *SE* = 0.66) than at the 0-ms SOA (*M* = 7.49%, *SE* = 0.82). The main effect of eye contact was not significant, *F*(1, 28) = 2.454, *p* = 0.128, *η*^2^_*p*_ = 0.081. The congruency × SOA interaction was significant, *F*(1, 28) = 52.660, *p* < 0.001, *η*^2^_*p*_ = 0.653. Paired t-tests indicated that errors were fewer on congruent than on incongruent trials at the 0-ms SOA, *t*(28) = 6.689, *p* < 0.001, *d* = 1.242, but not at the 900-ms SOA, *t*(28) = 1.983, *p* = 0.057, *d* = 0.368. No other significant results emerged (*F*s < 2.42, *p*s > 0.131), including the theoretically-relevant congruency × SOA × eye contact interaction (*F* < 1, *p* = 0.505; see Fig. 2).

#### Reflexive nature of saccades

Previous studies on overt gaze following reported that, on incongruent trials, saccades performed in the direction opposite to that indicated by the instruction cue and therefore erroneously driven by the gaze of the face stimulus (i.e., reflexive saccades) have shorter latencies as compared to saccades correctly executed in accordance with the instruction cue (i.e., voluntary saccades^33,46^). Here, we expected a similar pattern of results, with the additional hypothesis that eye contact episodes would increase the difference in saccadic latency between reflexive and voluntary saccades as compared to no eye contact episodes. Hence, mean latencies of reflexive and voluntary saccades made on incongruent trials (i.e., when the gaze of the face stimulus and the direction suggested by the instruction cue had opposite spatial vectors) were analysed. Because errors at the 900-ms SOA were modest, only saccades made at the 0-ms SOA were considered (see also^46^). Four participants could not be included in the analyses since they did not commit errors in two conditions (i.e., reflexive/eye contact and reflexive/no eye contact).

**Figure 3:**
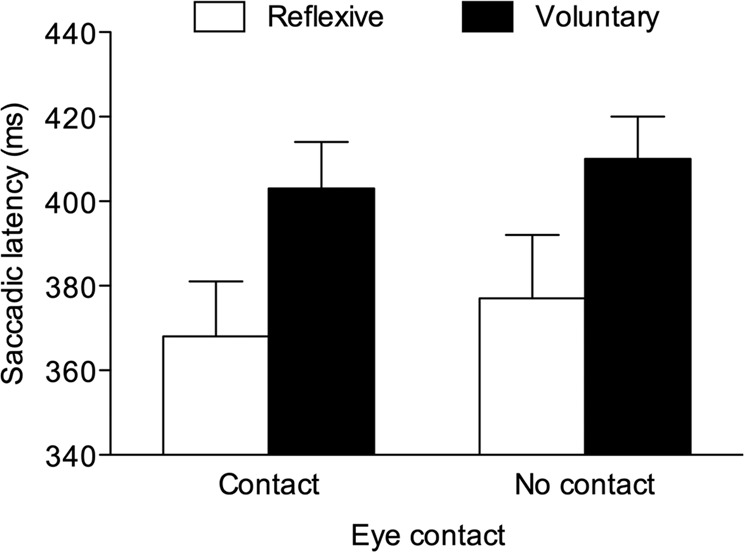
Experiment 1: Saccadic latencies for reflexive and voluntary saccades made on incongruent trials as a function of eye contact. Bars are SEM.

A repeated-measures ANOVA with saccade type (2: reflexive vs. voluntary) and eye contact (2: eye contact vs. no eye contact) as within-participant factors was carried out on mean saccadic latencies. The main effect of saccade type was significant, *F*(1, 24) = 23.277, *p* < 0.001, *η*^2^_*p*_ = 0.492, confirming that reflexive saccades had a shorter latency (*M* = 372 ms, *SE* = 12.74) than voluntary saccades (*M* = 407 ms, *SE* = 10.12). Both the main effect of eye contact (*F* = 1.239, *p* = 0.277) and the interaction between the two factors (*F* < 1, *p* = 0.875) were not significant (see also Fig. 3).

#### Relationship between overt gaze following and autistic-like traits

In order to unveil a possible relationship between autistic-like traits and the gaze following behaviour investigated here, the AQ scores were correlated with an overall index of gaze following magnitude. This index was computed as in a previous study investigating the potential link between autism and overt gaze following^46^. More precisely, saccadic latencies and accuracy were combined to obtain inverse efficiency scores^50,51^, computed separately for each combination of congruency and eye contact. More precisely, mean saccadic latencies were divided by the mean proportion of correct saccades. Then, the scores obtained on congruent trials were subtracted from those obtained on incongruent trials. Finally, the scores associated with eye contact trials were subtracted from those associated with no eye contact trials. The correlation analysis led to a non-significant result, *r* = 0.091, *p* = 0.640. Importantly, non-significant correlations also emerged when the eye contact (*r* = 0.200, *p* = 0.299) and the no eye contact (*r* = 0.295, *p* = 0.120) conditions were analysed separately.

#### Anticipatory saccades

Finally, we also tested whether eventual anticipatory saccades, resulting from the failure to maintain fixation, were more likely to occur right after eye contact episodes^46^. In more detail, we extracted the number of saccades made in the time window between averted-gaze onset and instruction cue onset, in order to explore the oculomotor behaviour before participants received the instruction to perform a saccade. This analysis was only possible for the 900-ms SOA condition since, at the 0-ms SOA, averted-gaze and instruction cue onsets were delivered simultaneously. The direction of saccadic eye movements was classified as either the same or different with respect to the spatial direction indicated by the distracting eye-gaze stimuli. Only 17 participants showed these anticipatory saccades. A repeated-measures ANOVA with spatial direction (2: same vs. different) and eye contact (2: eye contact vs. no eye contact) as within-participant factors was carried out on the mean number of saccades, but no significant result emerged (*F*s < 1.495, *p*s > 0.239).

#### Discussion

Overall, the main results emerging from this experiment suggest that a strong gaze following behaviour emerged at the shorter SOA, since shorter saccadic latencies and fewer errors were associated with congruent than incongruent trials. This pattern of results confirms the reliability of this task to investigate overt social attention mechanisms, in line with previous studies^32–34^. However, the magnitude of the overt gaze following behaviour, as well as other more related and fine-grained oculomotor markers, were not shaped by the eye contact manipulation. In Experiment 2, a variation in stimulus presentation was introduced as an attempt to make the presence of eye contact more salient.

## Experiment 2: Intermixed presentation of eye contact

Everything was identical to Experiment 1, with the only exception that the manipulation of eye contact was performed in an intermixed fashion. Indeed, there is evidence that specific information becomes more or less relevant as a function of the presence of a comparison item^52^. In this regard, previous work suggests that social modulations of gaze cuing can also be sensitive to the experimental context^53^. More precisely, Pavan and colleagues^53^ (Experiment 3) reported that gaze cuing was influenced by the group membership of the face providing the gaze, but only when this dimension was made salient in the experimental setting by presenting faces belonging to different social groups in an intermixed fashion. Thus, the modulation of social group on gaze-mediated orienting was visible when participants were induced to activate categorisation processes through social comparison (intermixed condition), but not when these processes were less likely to be triggered due to exposure to a single stimulus category (blocked condition). In a similar vein, here we reasoned that the expected modulatory effects of eye contact on overt gaze following might emerge through an intermixed presentation of this social episode, which would make the presence of eye contact much more salient than in the blocked presentation adopted in Experiment 1.

### Participants

A new sample of naïve students, with normal or correct-to-normal vision, was tested. Several booking slots were planned in advance, in order to at least achieve the sample size of Experiment 1. In so doing, when the testing session was ended, data of 45 participants (*Mean age* = 19 years, *SD* = 2.08, 9 males) were collected. All participants provided a written informed consent. The study was approved by the Ethics Committee for Psychological Research at the University of Padova and conducted in accordance with the Declaration of Helsinki.

### Apparatus

The apparatus was identical as that used in Experiment 1.

### Stimuli and procedure

Everything was identical to Experiment 1, with the following exception: Eye contact vs. no eye contact conditions were presented intermixed in the same blocks rather than in two separate blocks.

### Results

#### Data handling

Data were treated and analysed as in Experiment 1. Saccades with blinks were discarded (1.5% of trials). Saccadic directional errors were analysed separately (5.65% of trials). Outliers on correct trials were discarded from the analyses (0.64% of trials).

#### Saccadic latencies

**Figure 4:**
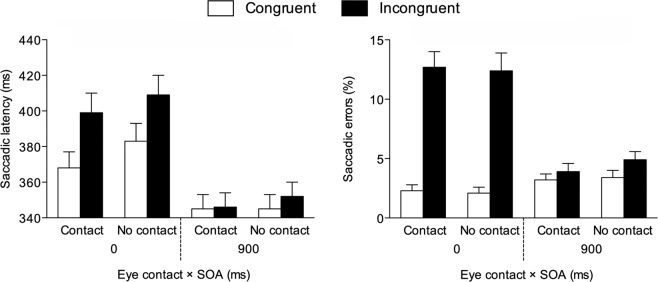
Experiment 2: Saccadic latencies and errors observed on congruent and incongruent trials as a function of SOA and eye contact. Bars are SEM.

Mean saccadic latencies were analysed through a repeated-measures ANOVA with congruency (2: congruent vs. incongruent), SOA (2: 0 vs. 900 ms) and eye contact (2: eye contact vs. no eye contact) as within-participant factors. The main effect of congruency was significant, *F*(1, 44) = 78.601, *p* < 0.001, *η*^2^_*p*_ = 0.641, due to shorter latencies on congruent (*M* = 360 ms, *SE* = 8.69) than on incongruent (*M* = 377 ms, *SE* = 9.13) trials, as well as the main effect of SOA, *F*(1, 44) = 117.874, *p* < 0.001, *η*^2^_*p*_ = 0.728, due to shorter latencies at the 900-ms SOA (*M* = 347 ms, *SE* = 8.18) than at the 0-ms SOA (*M* = 390 ms, *SE* = 9.9). Intriguingly, the main effect of eye contact was also significant, *F*(1, 44) = 18.486, *p* < 0.001, *η*^2^_*p*_ = 0.296, due to shorter latencies on eye contact trials (*M* = 365 ms, *SE* = 8.82) than on no eye contact trials (*M* = 372 ms, *SE* = 8.99). The congruency × SOA interaction was significant, *F*(1, 44) = 46.001, *p* < 0.001, *η*^2^_*p*_ = 0.511. Paired t-tests indicated that latencies were shorter on congruent than on incongruent trials both at the 0-ms SOA, *t*(44) = 8.945, *p* < 0.001, *d* = 1.333, and at the 900-ms SOA, *t*(44) = 2.170, *p* = 0.035, *d* = 0.324, but the difference between congruent and incongruent trials was smaller in the latter case (29 ms vs. 4 ms). The SOA × eye contact interaction was also significant, *F*(1, 44) = 12.602, *p* = 0.001, *η*^2^_*p*_ = 0.223. Paired t-tests indicated that latencies were shorter on eye contact than no eye contact trials at the 0-ms SOA, *t*(44) = 6.118, *p* < 0.001, *d* = 0.912, but not at the 900-ms SOA, *t*(44) = 1.214, *p* = 0.231, *d* = 0.181. No other significant results emerged, including the theoretically-relevant congruency × SOA × eye contact interaction (*F*s < 1.771, *p*s > 0.190; see Fig. 4).

#### Saccadic errors

The percentage of saccadic directional errors was analysed in the same manner as latencies. The main effect of congruency was significant, *F*(1, 44) = 45.990, *p* < 0.001, *η*^2^_*p*_ = 0.511, due to fewer errors on congruent (*M* = 2.77%, *SE* = 0.35) than on incongruent (*M* = 8.51%, *SE* = 0.86) trials, as well as the main effect of SOA, *F*(1, 44) = 40.942, *p* < 0.001, *η*^2^_*p*_ = 0.482, due to fewer errors at the 900-ms SOA (*M* = 3.89%, *SE* = 0.44) than at the 0-ms SOA (*M* = 7.39%, *SE* = 0.68). The main effect of eye contact was not significant (*F* < 1, *p* = 0.627). The congruency × SOA interaction was also significant, *F*(1, 44) = 57.577, *p* < 0.001, *η*^2^_*p*_ = 0.567. Paired t-tests indicated that errors were fewer on congruent than on incongruent trials both at the 0-ms SOA, *t*(44) = 7.542, *p* < 0.001, *d* = 1.124, and at the 900-ms SOA, *t*(44) = 2.165, *p* = 0.036, *d* = 0.323, but the difference between congruent and incongruent trials was smaller in the latter case (10.32% vs. 1.17%). The eye contact × SOA interaction was not significant (*F* = 1.429, *p* = 0.239). No other significant results emerged, including the theoretically-relevant congruency × SOA × eye contact interaction (*F*s < 1, *p*s > 0.526; see Fig. 4).

#### Reflexive nature of saccades

**Figure 5:**
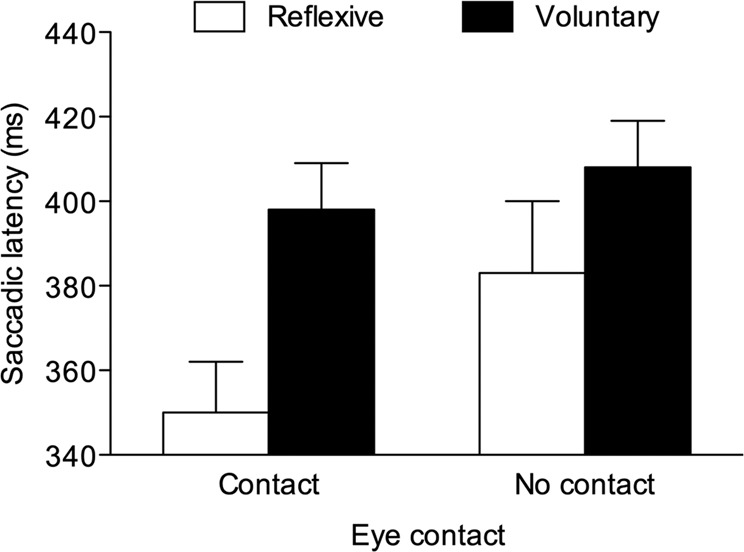
Experiment 2: Saccadic latencies for reflexive and voluntary saccades made on incongruent trials as a function of eye contact. Bars are SEM.

As in Experiment 1, only the 0-ms SOA condition was analysed. Three participants could not be included in the analysis since they did not commit errors in one condition (i.e., reflexive/eye contact). A repeated-measures ANOVA with saccade type (2: reflexive vs. voluntary) and eye contact (2: eye contact vs. no eye contact) as within-participant factors was carried out on mean saccadic latencies. The main effect of saccade type was significant, *F*(1, 41) = 20.658, *p* < 0.001, *η*^2^_*p*_ = 0.335, confirming that reflexive saccades had a shorter latency (*M* = 367 ms, *SE* = 13.56) than voluntary saccades (*M* = 403 ms, *SE* = 11.17), as well as the main effect of eye contact, *F*(1, 41) = 12.939, *p* < 0.001, *η*^2^_*p*_ = 0.240, due to shorter latencies on eye contact trials (*M* = 374 ms, *SE* = 10.76) than on no eye contact trials (*M* = 395 ms, *SE* = 13.37). Importantly, the saccade type × eye contact interaction was also significant, *F*(1, 41) = 4.600, *p* = 0.038, *η*^2^_*p*_ = 0.101. Paired t-tests indicated that reflexive saccades had shorter latencies than voluntary saccades after both eye contact, *t*(41) = 6.316, *p* < 0.001, *d* = 0.975, and no eye contact episodes, *t*(41) = 2.211, *p* = 0.033, *d* = 0.341, but the difference between reflexive and voluntary saccades was smaller in the latter case (48 ms vs. 25 ms; see also Fig. 5).

#### Relationship between overt gaze following and autistic-like traits

As in Experiment 1, the correlation between AQ and the difference in the inverse efficiency scores stemming from the eye contact and no eye contact conditions led to a non-significant result, *r* = −0.117, *p* = 0.444. This was true even when the eye contact (*r* = 0.111, *p* = 0.468) and the no eye contact (*r* = 0.010, *p* = 0.949) conditions were analysed separately.

#### Anticipatory saccades

Only 24 participants showed anticipatory saccades. A repeated-measures ANOVA with spatial direction (2: same vs. different) and eye contact (2: eye contact vs. no eye contact) as within-participant factors was carried out on the mean number of anticipatory saccades, leading to non-significant results (*F*s < 1).

#### Discussion

In line with Experiment 1, a reliable gaze following behaviour emerged in both saccadic latency and accuracy also in Experiment 2, and this was much more evident at the short SOA. However, unlike Experiment 1, in this context eye contact led to two crucial results. On the one hand, the eye contact condition was associated with an overall reduction of saccadic latencies. This is a relevant result which seems to indicate that our eye contact manipulation likely influenced the saccadic generation system. Furthermore, the overall reduction of saccadic latencies after eye contact episodes emerged at the short but not at the long SOA, confirming that the impact of social variables on social orienting may rapidly decay^21,38,48,49^. On the other hand – and more central to our hypotheses – eye contact also enhanced the reflexive component of overt gaze following, as indexed by a magnified difference in saccadic latencies for incongruent trials between reflexive and voluntary saccades after eye contact episodes.

## General discussion

The aim of the present study was to explore for the first time whether eye contact is able to enhance overt gaze following behaviour. In two experiments, a task-irrelevant central face either established eye contact with the participants or not, and then averted its gaze either leftwards of rightwards. Critically, the participants were asked to make leftwards and rightwards saccades in response to a central instruction cue, thus leading to both congruent conditions (i.e., the instruction cue and the eye-gaze stimulus indicated the same spatial location) and incongruent conditions (i.e., the instruction cue and the eye-gaze stimulus indicated opposite spatial locations). Overall, in both experiments evidence for a strong gaze following behaviour emerged, since faster saccadic latencies and less errors occurred on congruent than incongruent trials, replicating previous studies employing this task^32,33^. In both experiments, the magnitude of this pattern was not further modulated as a function of previous eye contact.

Nevertheless, in Experiment 2, eye contact had a critical impact on two different aspects of oculomotor behaviour. First, eye contact episodes were associated with an overall decrement in saccadic latencies. In other words, after eye contact, participants were overall faster in initiating a saccade as compared to the condition in which there were no previous eye contact episodes. Importantly, this effect associated to eye contact episodes was observed at the short (i.e., 0 ms) SOA but not at the long (i.e., 900 ms) SOA, in line with the view that the effects of task-irrelevant social variables on social orienting tend to be early-rising and short-lasting^21,38,48,49^. Second, the eye contact manipulation affected the reflexive component of overt gaze following. Indeed, on incongruent trials, saccades erroneously performed towards the location signalled by the averted-gaze face (i.e., reflexive saccades) had shorter latencies as compared to saccades correctly performed in accordance with the instruction cue (i.e., voluntary saccades^33^), and the difference between reflexive and voluntary saccades was greater after eye contact episodes. The fact that significant effects of eye contact emerged only when the eye contact manipulation was presented intermixed, rather than blocked, seems to suggest that this phenomenon is highly sensitive to contextual factors, such as the presence of different types of facial stimuli along the critical dimension within the same block of trials, thus aligning with previous research exploring the role of racial group membership on covert orienting^53^. In Experiment 2, the presence of faces either establishing eye contact or not with the participants in the same block of trials was more likely to render this task-irrelevant dimension contextually salient. In contrast, when eye contact conditions were presented in different blocks (Experiment 1), the lack of variability in stimulus presentation within each block made participants less likely to focus on whether the face had established eye contact or not. In other words, the presence of eye contact becomes more or less relevant as a function of the presence of a comparison item (i.e., a face that does not look at us; see^52^).

The observation that, in Experiment 2, eye contact led to an overall decrement in saccadic latencies resembles the pattern described in a recent study reporting that participants reacted more readily to peripheral targets after making eye contact with a real individual^54^. According to the authors, this might be due to the enhanced levels of arousal that are typically elicited by eye contact episodes which, in turn, would lead participants to be more prone to react to the onset of visual stimuli. Although some evidence suggests that arousal enhancements are especially likely after eye contact with real individuals^6^, they have also been reported using pictorial stimuli of direct-gaze faces when participants have to perform a high demanding task^11^. In this regard, we can speculate that the instructed saccade task employed here was indeed sufficiently demanding to reveal modulations of saccadic latencies due to eye contact.

The main finding of a magnified difference in saccadic latencies between reflexive and voluntary saccades after eye contact episodes is in line with the available evidence with manual responses and covert orienting^27–29,55^. Our study extends and qualifies previous work using more direct measures and a paradigm with higher ecological validity. Indeed, eye movements represent the major route through which we sample relevant information from the environment, and oculomotor measures provide explicit markers of attentional allocation over space.

Another finding emerging from this work is the lack of a relationship between autistic-like traits, assessed through the AQ^41^, and the overall magnitude of overt gaze following behaviour (see also^16,46^). In this regard, it should be stressed that non-clinical individuals took part in the present experiments. Future studies might explore whether clinical samples show the same trend.

Overall, this study provides novel evidence confirming the relevance of eye contact in modulating human behaviour, at least under conditions that promote its contextual saliency, and aligns with the notion that eye contact is capable of amplifying different cognitive and physiological mechanisms^6–10^. In this regard, the fact that eye contact enhanced the reflexive component of overt gaze following fits nicely with the “fast track modulator” model pushed forward by Senju and collaborators^8,9^. This model states that direct-gaze stimuli would be initially processed by a dedicated subcortical neural network involving the superior colliculus (SC). Interestingly, several studies identified in the SC one of the main generators of saccadic eye movements^56^, and this would be particularly the case for those produced reflexively^57^. Hence, the enhanced reflexive orienting emerged in Experiment 2 might reflect an enhanced SC activity likely elicited by direct-gaze stimuli. This reasoning might find support in the observation that the influence of eye contact emerged at the short SOA only, namely in a condition in which top-down mechanisms grounded on cortical substrates were much less likely to be involved.

To conclude, this work provides supporting evidence for the notion that the reflexive component of overt gaze following can be enhanced after eye contact, confirming that this type of social stimulus is able to boost the mechanisms underlying social orienting. Establishing eye contact is a key factor in social life, signalling that potential interaction episodes are likely to occur. Overall, we tend to more likely follow the gaze of individuals that are subjectively appraised as more similar and relevant to us^35,38^. The enhanced gaze-following behaviour after eye-contact indicates that we are strongly engaged by individuals looking into our eyes, thus making the attentional behaviour signalled by their gaze shifts maximally relevant for us.

## Data Availability

The datasets generated and analysed during the current study are available from the corresponding author on reasonable request.
